# Multi‐century trends to wetter winters and drier summers in the England and Wales precipitation series explained by observational and sampling bias in early records

**DOI:** 10.1002/joc.6208

**Published:** 2019-07-11

**Authors:** Conor Murphy, Robert L. Wilby, Tom K. R. Matthews, Peter Thorne, Ciaran Broderick, Rowan Fealy, Julia Hall, Shaun Harrigan, Phil Jones, Gerard McCarthy, Neil MacDonald, Simon Noone, Ciara Ryan

**Affiliations:** ^1^ Irish Climate Analysis and Research UnitS (ICARUS), Department of Geography Maynooth University Maynooth Ireland; ^2^ Department of Geography and Environment Loughborough University Loughborough UK; ^3^ Institute of Hydraulic Engineering and Water Resources Management Technische Universität Wien Vienna Austria; ^4^ European Centre for Medium‐Range Weather Forecasts (ECMWF) Reading UK; ^5^ Climatic Research Unit University of East Anglia Norwich UK; ^6^ School of Environmental Sciences University of Liverpool Liverpool UK

**Keywords:** data quality, drier summers, England Wales Precipitation, Gordon Manley, historical climate, sleet and snow, wetter winters

## Abstract

Globally, few precipitation records extend to the 18th century. The England Wales Precipitation (EWP) series is a notable exception with continuous monthly records from 1766. EWP has found widespread use across diverse fields of research including trend detection, evaluation of climate model simulations, as a proxy for mid‐latitude atmospheric circulation, a predictor in long‐term European gridded precipitation data sets, the assessment of drought and extremes, tree‐ring reconstructions and as a benchmark for other regional series. A key finding from EWP has been the multi‐centennial trends towards wetter winters and drier summers. We statistically reconstruct seasonal EWP using independent, quality‐assured temperature, pressure and circulation indices. Using a sleet and snow series for the UK derived by Profs. Gordon Manley and Elizabeth Shaw to examine winter reconstructions, we show that precipitation totals for pre‐1870 winters are likely biased low due to gauge under‐catch of snowfall and a higher incidence of snowfall during this period. When these factors are accounted for in our reconstructions, the observed trend to wetter winters in EWP is no longer evident. For summer, we find that pre‐1820 precipitation totals are too high, likely due to decreasing network density and less certain data at key stations. A significant trend to drier summers is not robustly present in our reconstructions of the EWP series. While our findings are more certain for winter than summer, we highlight (a) that extreme caution should be exercised when using EWP to make inferences about multi‐centennial trends, and; (b) that assessments of 18th and 19th Century winter precipitation should be aware of potential snow biases in early records. Our findings underline the importance of continual re‐appraisal of established long‐term climate data sets as new evidence becomes available. It is also likely that the identified biases in winter EWP have distorted many other long‐term European precipitation series.

## Graphical abstract

The trend towards wetter winters and drier summers in EWP can be explained by changes in measurement practice and data biases. For winter (Figure below), the timing of divergence aligns with the introduction of the Snowdon Pattern Rain Gauge in the UK around 1863. Given the widespread use of EWP across environmental and climate research, we expect that our findings will challenge understanding of multi‐centennial trends in precipitation in the UK. These changes in measurement practice are also likely to affect other long‐term precipitation records in north‐west Europe.

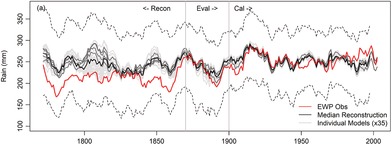

## 1 INTRODUCTION

Long‐term, quality‐assured records are essential for understanding climate variability and change (Brázdil *et al.,* 2005). Globally, few such records extend to the 18th century, particularly for precipitation. The England Wales Precipitation (EWP) series (Wigley *et al.,* 1984; Wigley and Jones, 1987; Alexander and Jones, 2000; Simpson and Jones, 2014) is a rare exception, providing a continuous monthly record from 1766. This areal precipitation series, derived from five rainfall regions to avoid sampling bias, is regarded as homogenous (Wigley *et al.,* 1984; Croxton *et al.,* 2006) and is routinely updated by the UK Met Office (UKMO) Hadley Centre. Over more than three decades, EWP has found widespread use in understanding precipitation variability in Northwest Europe (e.g., Murphy *et al.,* 2018), trend detection (Gregory *et al.,* 1991; Jones *et al.,* 1997; Jones and Conway, 1997; Jenkins *et al.,* 2008; Kendon *et al.,* 2017; 2018), evaluation of climate model simulations (Dessai and Hulme, 2008), as a proxy for mid‐latitude atmospheric circulation (Luterbacher *et al.,* 2001), a predictor in long‐term European gridded precipitation data sets (Pauling *et al.,* 2006; Casty *et al.,* 2007), the assessment of droughts (Marsh *et al.,* 2007) and extremes (Beran, 2002), tree‐ring reconstructions (Rinne *et al.,* 2013) and as a benchmark for other regional series (Noone *et al.,* 2016).

A key finding from analysis of EWP is a trend towards wetter winters (DJF) and drier summers (JJA) (Gregory *et al.,* 1991; Jones *et al.,* 1997; Jones and Conway, 1997; Jenkins *et al.,* 2008; Kendon *et al.,* 2017). This is consistent with regional climate model projections for coming decades (Maisey *et al.,* 2018). However, both trends depend critically on the early series, prior to the pioneering work of George J. Symons and Charles Higman Griffith who, in the 1860s, led the standardization of rain gauge design, exposure and measurement practice in the UK (Walker, 2010; Burt, 2013). Despite widespread application of EWP over more than three decades, the early record and associated trends have not been re‐evaluated in light of modern evidence, while homogeneity issues have been recognized at constituent gauges (Wigley *et al.,* 1984; Burt and Howden, 2011). Here, we statistically reconstruct EWP using independent, quality‐assured temperature, pressure and circulation indices to examine the early record.

## 2 DATA AND METHODS

Monthly EWP data were accessed from the UKMO (https://www.metoffice.gov.uk/hadobs/hadukp/) and the winter (DJF), spring (MAM), summer (JJA) and autumn (SON) series compiled. Winters are specified by the year in which January falls. We reconstruct EWP seasonal precipitation using multiple linear regression. Emphasis is placed on the winter (henceforth *EWP*_*w*_) and summer (henceforth *EWP*_*s*_) series given the previously identified trends in these seasons.

Predictors are based on available long‐term observed and reconstructed sea level pressure (SLP), temperature and wind direction series (listed in Table 1 and described below). Predictor selection and estimation of model coefficients were undertaken for the calibration period 1900–2002; the period of optimum overlap between EWP and predictors. Simulations were evaluated for the years 1870–1899, before focusing attention on our reconstruction period 1767–1869 (i.e., before the work of Symons and Higman Griffith).

**Table 1:** Overview of predictors and associated data used for model building, together with their relation to EWP

Predictor data sets and period used	Relation to EWP
CET 1767–2002 (Manley, 1974; Parker *et al.,* 1992)	Warm winters tend to be wetter winters through enhanced advection.
WI 1767–2002 (Barriopedro *et al.,* 2014); Wheeler *et al.,* 2010)	Measure of the persistence of westerly winds beneath the exit zone of the North Atlantic extratropical jet stream.
PL 1767–2002 (Cornes *et al.,* 2013)	Measure of westerly air flow over Northwest Europe. Used as an indicator of the state of the NAOI.
LSLP 1767–2002 (Cornes *et al.,* 2012a)	MSLP for the city of London. High pressure is associated with lower precipitation totals.
Leading EOF of reconstructed gridded SLP (KEOF) 1767–2002 (Küttel *et al.,* 2010)	Surrogate for the NAOI from independent SLP reconstructions
Average of correlated grids from reconstructed gridded SLP (KAVG) 1767–2002 (Küttel *et al.,* 2010; Baker *et al.,* 2018)	SLP for area representing 50^o^–60^o^N, 10^o^W–5°E high pressure associated with lower precipitation totals.

*Note*: Full details of each predictor are provided in the Section 2.

### 2.1 Predictor variables

Long‐term predictor variables that are independent from EWP and describe important dynamics of regional precipitation were available for model building. While these have been independently quality assured, we acknowledge that each may be subject to unknown inhomogeneities. Our predictors were:

- *Central England Temperature (CET)*: (Manley, 1974; Parker *et al.,* 1992). Previous research shows that CET winter and summer temperatures are strongly correlated with precipitation in the British and Irish Isles (Murphy *et al.,* 2018). CET data were downloaded from the UKMO (https://www.metoffice.gov.uk/hadobs/hadcet/) and seasonal mean temperature extracted.
- *Westerly Index (WI)*: A monthly index of atmospheric circulation variability over the North Atlantic from 1685–2008 (Barriopedro *et al.,* 2014), based on direct observations of wind direction from Royal Navy logbooks from 1685 to 1850 provided by ship movements in the English Channel. After 1850, the CLIWOC v1.5 (García‐Herrera *et al.,* 2005) and the ICOADS v2.1 (Worley *et al.,* 2005) data sets are used. The WI provides a measure of the persistence of westerly winds beneath the exit zone of the North Atlantic extratropical jet‐stream roughly covering the English Channel area (Wheeler *et al.,* 2010). Before 1850, the WI contains only one record of wind direction (measured with a 32‐point compass) per day, with data available for 95% of days (Barriopedro *et al.,* 2014). The monthly WI used here is defined as the percentage of days per month with prevailing wind from the west (i.e., blowing from between 225 and 315° from true north).
- *London Sea Level Pressure (LSLP)*: A 315‐year (1692–2007) daily series of mean SLP (MSLP) for the city of London (Cornes *et al.,* 2012a). Digitized data were transcribed from multiple sources, quality controlled, corrected and homogenized to represent daily means of MSLP at standard modern‐day conditions (Cornes *et al.,* 2012a). Monthly values of MSLP are not reported when missing values exceed 20%. Data were obtained from the Climatic Research Unit (CRU) at the University of East Anglia (https://crudata.uea.ac.uk/cru/data/parislondon/).
- *Paris London Index (PL)*: An indicator of the state of the North Atlantic Oscillation Index (NAOI) over the years 1692–2007, providing a consistent measure of westerly airflow over Northwest Europe (Cornes *et al.,* 2013). The index was developed from MSLP data recovered and corrected for the respective cities (Cornes *et al*., 2012a; 2012b). Data were obtained from CRU (https://crudata.uea.ac.uk/cru/data/parislondon/).
- *Küttel EOF*: Küttel *et al*. (2010) extended the HadSLP2 data set (1850–2004) (Allan and Ansell, 2006) to 1750 using a combination of homogenized instrumental pressure series and data from ship log books derived from the CLIWOC data set for years up to 1855 and wind information from the ICOADS database V2.4. Reconstructions are presented as a 5 × 5° resolved gridded seasonal SLP data set covering the eastern North Atlantic, Europe and the Mediterranean area (40°W–50°E; 20°–70°N) back to 1750. Data were downloaded from the NOAA National Centres for Environmental Information National Climatic Data Centre (ftp://ftp.ncdc.noaa.gov/pub/data/paleo/historical/kuettel2009slp.txt). We derived the first Empirical Orthogonal Function (EOF) of the gridded SLP (KEOF) as a measure of the NAOI.
- *Küttel AVG*: We use the Küttel *et al*. (2010) gridded SLP reconstructions to derive winter and summer average SLP predictors for a grid box representing the UK. Following previous work, we define *Küttel AVG* as the standardized mean MSLP anomaly in a box centred over the UK (50°–60°N, 10°W–5°E) (Baker *et al.,* 2018).
- *Küttel Grids*: We also identify individual 5 × 5° grids that provide significant predictors of EWP_w_ and EWP_s_. These grids are numbered using the naming convention of the original data set (Küttel *et al.,* 2010). Grid point 1 is centred at 40°W/70°N, grid point 2 at: 35°W/70°N, through to grid point 209 centred at 50°E/20°N.

### 2.2 Model selection, fitting and testing for seasonal EWP

Multiple linear regression models were developed for each season with predictors selected during the calibration period 1900–2002 (the maximum overlap for available predictors as gridded SLP reconstructions end in 2002). Both the predictand (observed EWP) and potential predictors were checked for compliance with the assumptions of ordinary least squares regression. Winter and autumn CET were found to be non‐normally distributed, so were transformed using a Box‐Cox Transformation (estimated Lambda 1.4 and 1.2, respectively) (Osborne, 2010). Observed autumn EWP was also found to be non‐normal (*p* = .04), however, for ease of interpretation and plotting was not transformed. Models were selected for use provided they: (a) explain more than 70% of variance (*R* ^2^) in winter and summer EWP and more than 60% in spring and autumn EWP over the full calibration period; (b) predictors are a statistically significant (*p* < .05) addition to the model and (c) are unaffected by multicollinearity (indicated by the Variance Inflation Factor).

To explore the effect of uncertainty in calibration data on simulations, selected models were fitted using 1,000 bootstrap re‐samples of 50 years (with replacement) from the calibration data set. These re‐samples were then used to establish (a) a median simulation for each selected model; (b) an ensemble median simulation across all models and (c) 95% confidence intervals across all models. All resampled simulations were assessed to ensure the *iid* assumption of OLS residuals. Simulations were evaluated using independent data for the period 1870–1899 before examining reconstructions for the period 1767–1869. Decadal means were derived using a centred moving average.

### 2.3 Investigating winter model residuals

Manley's London sleet and snow (SS) series was used to investigate the potential role of snow under‐catch on EWP_w_ model residuals. The full SS series runs from 1668–1974. These data were originally tabulated by Gordon Manley and Elizabeth Shaw drawing on numerous gauges in the greater London area (Manley, 1969). The data are assumed to represent an elevation up to 200 ft (Manley, 1969). While plotted and discussed in Manley's original 1969 paper, the data were never digitized. Following his death in 1980, Manley's notes were deposited by Mrs. Audrey Manley (his widow) in Cambridge University Library. These are housed in a dedicated archive (Reference: GB 12 MS.Add.8386) with his notes on snow contained in Box 16/27. These notes remain in the original envelopes in which they were deposited. The lead author visited this archive in December 2017 and scanned the data relating to the London SS series. These were later transcribed using double keying to avoid data entry errors.

Manley subjected the series to substantial quality control. The series is never based on fewer than three good quality stations back to 1811 and at least two stations between then and the 1680s (Manley, 1969). Correction was made for the adoption of the Gregorian calendar in September 1752. Observations of the occurrence of sleet and snow were categorized into four quality grades based on the assumed “alertness” of the observer: A – data from first‐class airfields and observatories; B – data from keen climatological observers; C – data from sites at which one daily visit was made to the instruments and D – data from stations overseen by observers who had other duties (Manley, 1969). Table S1 provides a list of the stations contributing to the London SS series, together with their altitude, observer surname and quality control grade assigned by Manley to the data. Comparisons of model residuals and the London SS series are undertaken using centred decadal means. Where correlations are presented, they are derived using Pearson's correlation.

### 2.4 Additional models developed

Regression models are also derived for selected constituent EWP series. In order to aid the interpretation of EWP_s_ model residuals, reconstructions of summer precipitation at Kew and Oxford were derived. Both stations are influential constituents of EWP for the south east England region and among the longest available series in the UK. Precipitation totals for both stations were obtained from CRU (https://crudata.uea.ac.uk/cru/data/UK_IR_rainfall_data/) and summer (JJA) totals extracted. The same predictors and study design as above were used for reconstructing both series, with the exception that only the best fit model was employed for each station.

### 2.5 Testing for trends

Evidence of monotonic trends in observed and simulated EWP and Manley's London Sleet and Snow Series was assessed using the non‐parametric Mann–Kendall (MK) test (Mann, 1945; Kendall, 1975). While a monotonic trend is likely a poor model for UK precipitation, such a model has nonetheless been widely applied. Here, we use the MK test primarily as an analytical tool to examine differences between observations and reconstructions. The standardized MK statistic (MKZs) follows the standard normal distribution with a mean of zero and variance of one. A positive (negative) MKZs indicates an increasing (decreasing) trend. Statistical significance was evaluated with probability of type 1 error set at the 5% significance level. We apply a two‐tailed MK test; hence, the null hypothesis of no trend (increasing or decreasing) is rejected when |*MKZs*| > 1.96. All variables were checked for positive lag‐1 serial correlation at the 5% level using the Durbin Watson test. The MK test was applied to simulated and observed EWP for different time periods (with at least 30 years) to examine consistency of trends depending on start and end years. To examine robustness of trends ending in 2002, the MK test was also applied to the full observed *EWP*_*w*_ and *EWP*_*s*_ series ending in 2018. For Manley's London SS series the MK test was applied to the full series 1669–1974.

## 3 RESULTS

### 3.1 Winter

**Figure 1:**
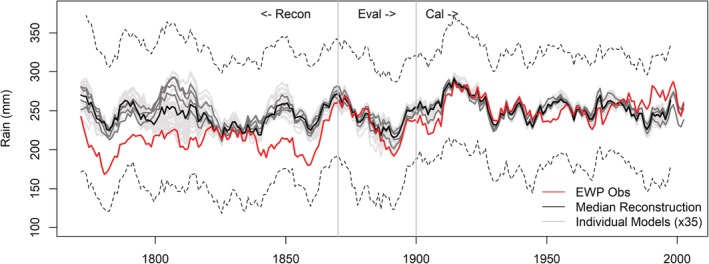
Decadal mean observed and modelled *EWPw* 1767–2002. Included are median simulations from each of the 35 individual models (grey lines) together with the ensemble median (black line). Dark grey lines represent models based on observed data only. Dashed lines indicate upper and lower 95% confidence intervals for reconstructions

We identify 35 models for simulating *EWP*_*w*_ (Table S2). Models are skilful and show equally good performance for calibration and evaluation periods (Table S2). During the period 1767–1869, *EWP*_*w*_ is consistently lower than the median simulation, except for the period 1825–1840 (Figure 1). Notably, simulations show higher uncertainty for the period 1790–1825, with lower bound simulations falling close to observed *EWP*_*w*_. Closer inspection reveals exceptional zonal differences in mean SLP for these decades (Figure S1), which were remarkable in the full record of EWP (Figure S2). Thirty‐year (1791–1820) mean SLP anomalies show higher than average pressure in the eastern Atlantic and lower than average pressure over the UK and Europe. This is consistent with prevalence of strongly negative NAO conditions during this time, also evident in the WI, KEOF and PL indices. Quality assured observations of SLP at London (LSLP), Edinburgh and Paris confirm the tendency for lower than average SLP over the region during this period (Figure S3) and that gridded reconstructions underestimate SLP. Simulations lying close to observed *EWP*_*w*_ during the early 1800s (Figure 1) originate from reconstructed SLP grids located west of the UK (higher than average pressure). Based on the above evidence these grids are regarded as unrepresentative of UK conditions at that time, so were removed from further analysis.

Lower than expected observed *EWP*_*w*_ precipitation totals during our reconstruction period are unlikely reflecting changing station density in EWP given the relatively stable number of stations in EWP throughout most of the 19th century (Wigley *et al.,* 1984). Although colder winters tend to be drier, we find a bias in the pre‐1870 period, whereby precipitation totals during colder than average (1766–2002) winters are significantly lower than expected when compared with the post‐1870 record (*t* test −2.39; *p* < .05). From early observations at well documented stations it is unclear whether observers were actually measuring snowfall (Smith, 1979; Burt and Howden, 2011). Moreover, in an address to the Royal Meteorological Society in 1891, Symons noted that prior to the introduction of the Snowdon pattern rain gauge in 1864, gauges were subject to large under‐catch during snowfall due to the absence of a protective rim. He asserted that “most of the early English records are (for the winter months) too small [low], owing to insufficient attention to the measurement of snow” (Symons, 1891).

**Figure 2:**
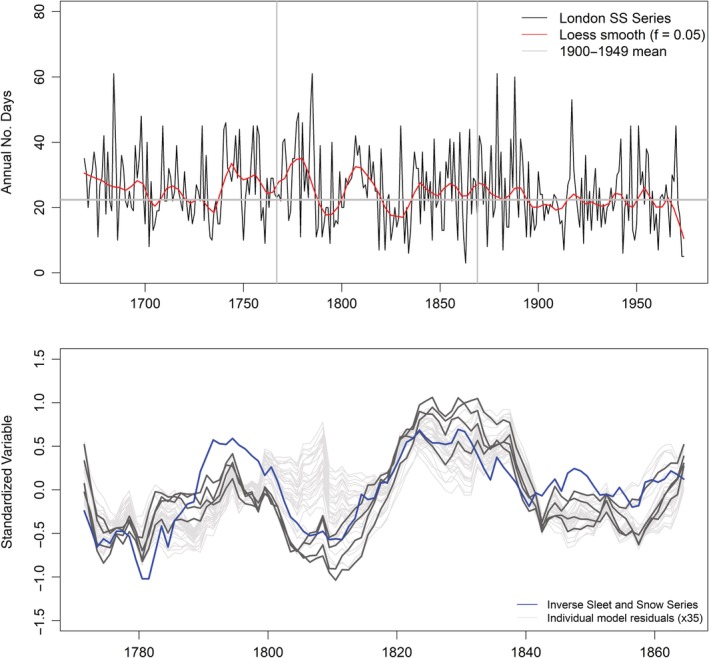
(a) Manley's Sleet and Snow Series for the greater London area 1669–1974. Also plotted is the loess smoothed (*f* = 0.05) series. Vertical grey lines indicate years employed to investigate winter model residuals. (b) Comparison of standardized decadal mean inverse sleet and snow series (blue) 1767–1869 with standardized decadal mean model residuals. Grey lines represent median residuals from each model, dark grey lines are models based on observations only. Black line shows the ensemble median residual

We employ the sleet and snow (henceforth SS) series compiled by Gordon Manley and Elizabeth Shaw (Manley, 1969) to investigate this bias. The series exhibits a significant decreasing trend in the annual frequency of SS days (MKZs −2.68; *p* < .05) (Figure 2a). During our reconstruction period (1767–1869) persistent periods of high SS frequency, in excess of the 1900–1949 mean, are apparent (Figure 2a). Also evident is that the period 1825–1840 (years for which *EWP*_*w*_ and reconstructions agree) shows low SS frequency–lower even than the 1900–1949 mean (Figure 2a). Notably, the SS series explains between 25 and 66% of the variance in decadal *EWP*_*w*_ model residuals (Figure 2b); (48–66% for models based on observed SLP). Unexplained variance is likely associated with a combination of non‐standard gauge heights (above the ground) in the early series and associated wind loss (Craddock, 1976), together with under‐representation of upland gauges in the early EWP series (Wigley *et al.,* 1984).

**Figure 3:**
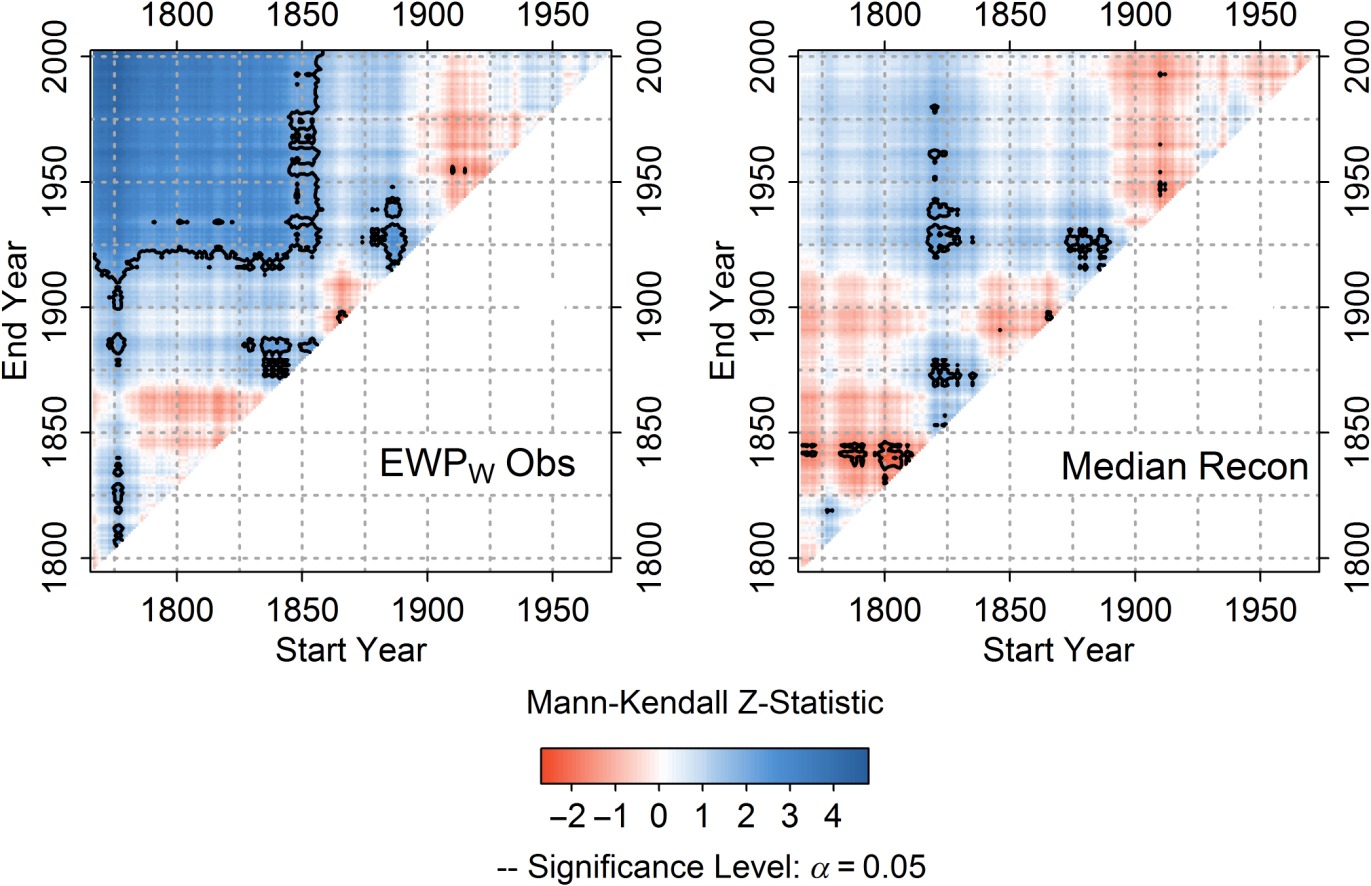
Mann Kendall Zs scores for observed *EWPw* and median model reconstruction for all combination of start and end years (minimum 30‐years) for the period 1767–2002. Black contour marks significant trends (*p* < .05)

Investigation of monotonic trends in observed and simulated *EWP*_*w*_ reveals large differences in the strength and significance of trends. Observed EWP_w_ shows highly significant increasing trends for tests commencing before 1860 (Figure 3). Significant trends for the equivalent period are not evident in the median reconstruction (Figure 3). Inspection of trends across all models (Figure S4) reveals that for the full period of analysis (1767–2002), 94% of simulations show no significant increasing trend in *EWP*_*w*_. Of the simulations that show significant tends, the magnitude is substantially less than observed. For later periods (Figure S4b,c) trends in modelled and observed *EWP*_*w*_ are more consistent, whereby both simulated and observed *EWP*_*w*_ reveal non‐significant changes.

### 3.2 Summer

**Figure 4:**
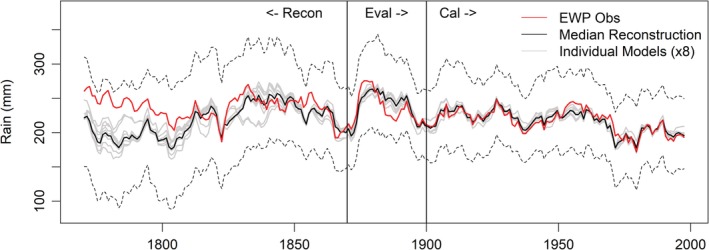
Decadal mean observed and modelled *EWPs* 1766–2002. Included are median simulations from each of the eight individual models (grey lines) together with the ensemble median (black line). Dashed black lines indicate the upper and lower 95% confidence intervals for reconstructions

We identify eight models for simulating *EWP*_*s*_ (Table S3). Models show equally good skill for calibration and evaluation periods (Table S3). For the period prior to 1820, *EWP*_*s*_ is consistently above the median simulation (Figure 4). Critically, there are fewer stations contributing to EWP prior to 1820 (Wigley *et al.,* 1984). From 1789–1819 the number of stations per region falls to at most two, with occasionally some regions containing no stations and others only one. Prior to 1789, there is only one station per region used to derive EWP (Wigley *et al.,* 1984). Thus, changes in network density may mis‐represent true summer precipitation, with the bias dependent upon whether retained stations preferentially sample wetter or drier regions.

**Figure 5:**
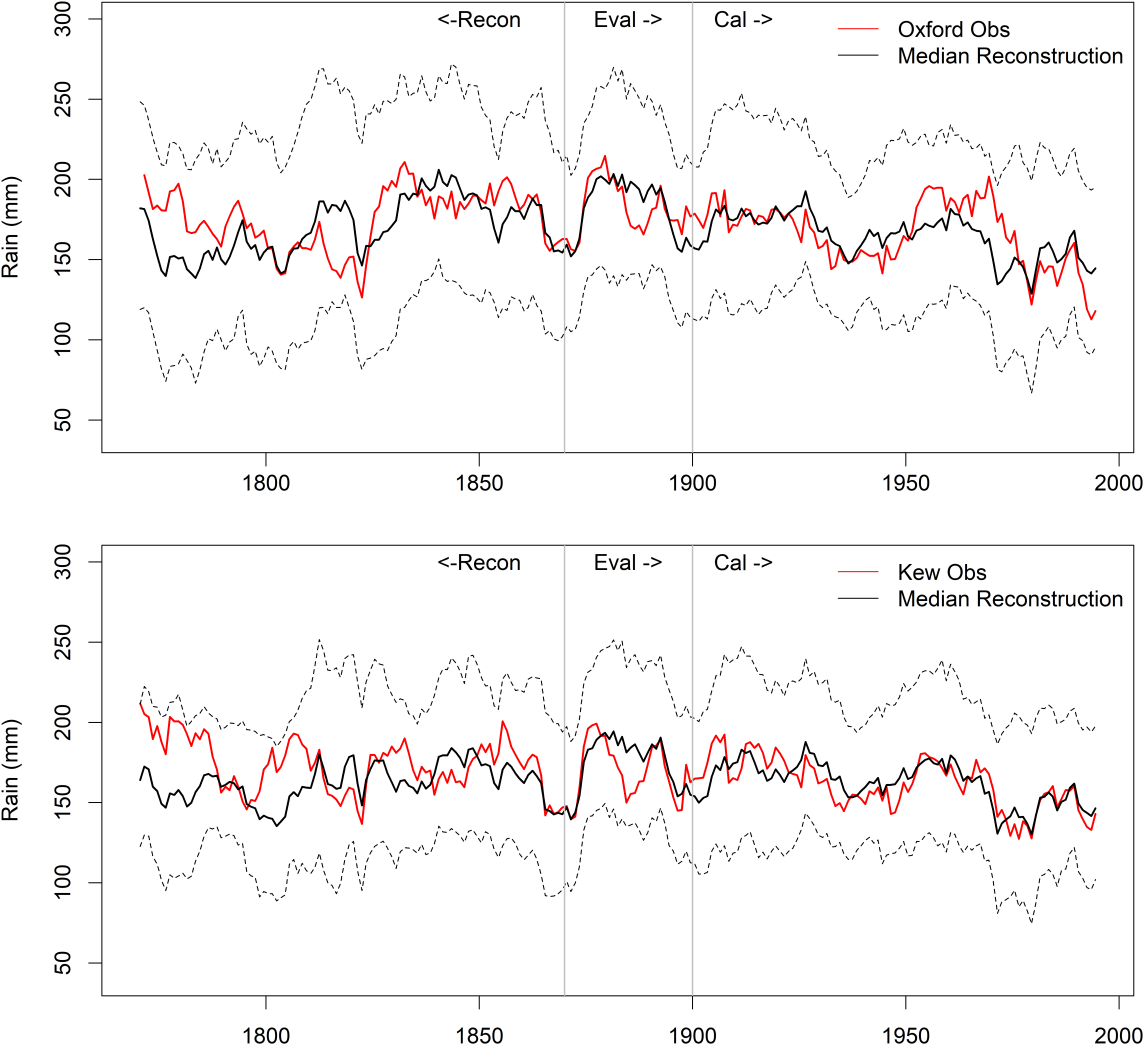
Decadal means for observed and modelled Oxford (top) and Kew (bottom) summer (JJA) precipitation respectively. The median reconstruction is shown by the black line, the observed totals in red. Dashed black lines indicate the upper and lower 95% confidence intervals for reconstructions

Second, many of the early records for key stations (e.g., Oxford, Kew) are composites of several series, often with inconsistent (sometimes unknown) gauge designs and exposures across sites. For example, observations for Oxford prior to 1815 are fragmentary and derived from various London stations and weather diaries, and thus associated with greater uncertainties (Craddock and Craddock, 1977). Similarly, early records for Kew are based on stations from the London area (Wales‐Smith, 1971). Reconstruction of both series for summer shows similar overestimation to that revealed for *EWP*_*s*_ reconstructions (Figure 5). The over‐catch evident in the early records of both series is robust to the choice of predictors. It should be noted that *R* ^2^ values for the derived Kew and Oxford models are lower than those for the EWP regional series (Table S4).

**Figure 6:**
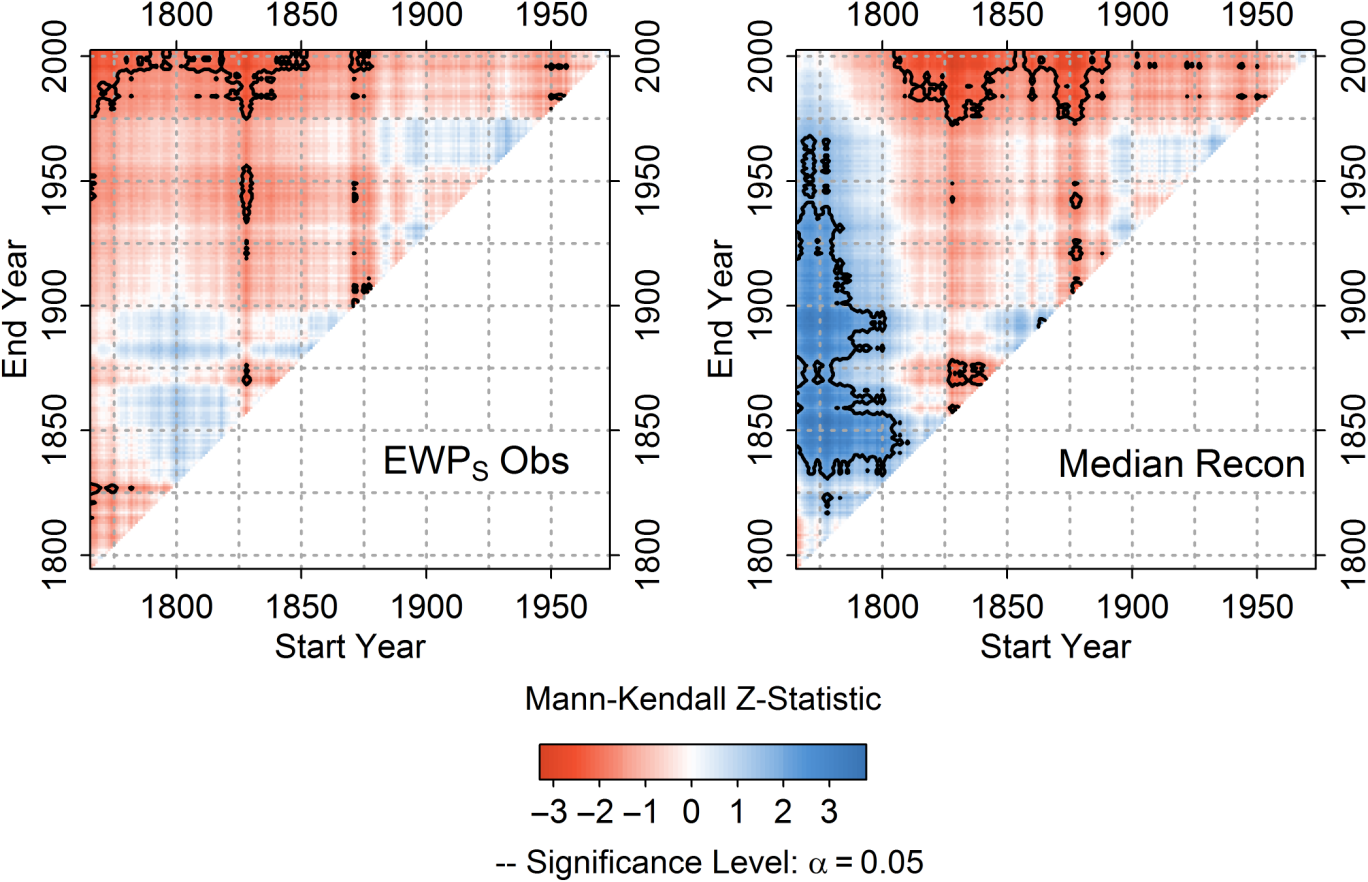
As in Figure 3 but for *EWPs* and the median model reconstruction

Observed *EWP*_*s*_ exhibits a significant decreasing trend over the full record analysed (1766–2002) (*MKZs*: −2.81; *p* < .05) (Figure 6). This decline is not evident in the median reconstruction (Figure 6). Across all models most simulations (>99%) show weak, non‐significant trends for the full period of analysis (1766–2002), emphasizing the importance of the early series to the derived trend (Figure S5). Significant decreasing trends are evident from reconstructions for tests commencing during the 1800s (Figure 6 & Figure S5), consistent with archival evidence of flood rich summers at that time (Macdonald, 2012). However, significant decreasing trends are only derived from models that use *reconstructed* SLP. Simulations based on *observed* SLP (which have been rigorously quality assured) show no such evidence of significant decreasing trends and are more consistent with trends in observed *EWP*_*s*_ (Figure S5).

### 3.3 Spring and autumn

**Figure 7:**
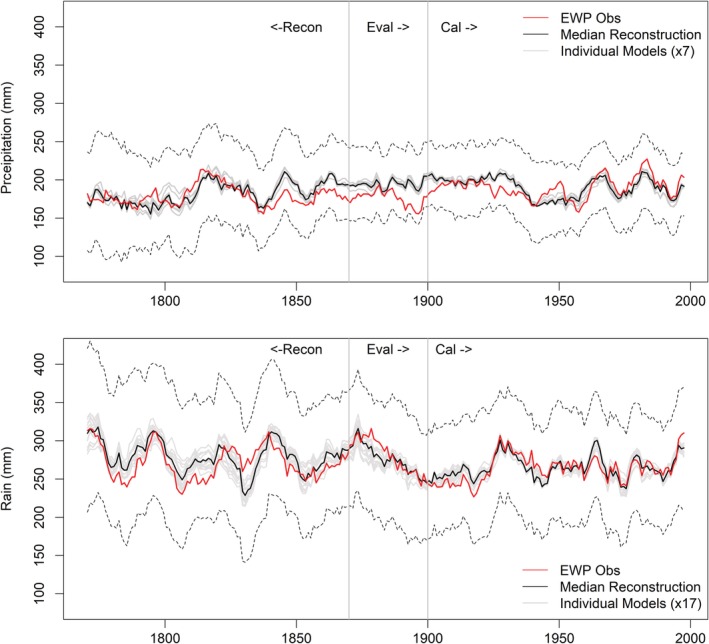
Decadal rolling means for observed and modelled EWP spring (MAM) top and autumn (SON) bottom for years 1766–2002. Included are median simulations from each identified model (grey lines) together with the ensemble median (black line). Dashed black lines indicate the upper and lower 95% confidence intervals for reconstructions

In total seven models were identified for simulating *EWP*_*sp*_ and 17 models for *EWP*_*au*_. Regression model results are presented in Tables S5 and S6, respectively. For both seasons, there is good agreement between observations and reconstructions throughout the record (Figure 7). For autumn, however, there is some evidence that observed *EWP*_*au*_ is too low during periods of high sleet and snow frequency at the start of the 19th Century, and in the 1770s. While the magnitude of underestimation is considerably less than for *EWP*_*w*_, the London SS series explains up to 20% of the variance in decadal *EWP*_*au*_ model residuals for the pre‐1870 reconstruction period. For *EWP*_*sp*_ we find no discernible relationship between model residuals and the SS series.

## 4 CONCLUSIONS

Our analysis reveals significant discrepancies between reconstructed and observed *EWP*_*w*_ and *EWP*_*s*_ in the pre‐1870 record. For *EWP*_*w*_, the timing of divergence aligns with the introduction of the Snowdon Pattern Rain Gauge (Smith, 1979). We show that the widely accepted trend to wetter winters in observed *EWP*_*w*_ is likely an artefact of measurement practice and (probably) higher occurrence of snowfall in early winters. However, rising UK temperatures over the last two centuries (Kendon *et al.,* 2018) have also influenced *EWP*_*w*_ by altering the phase of winter precipitation, as evidenced by a significant decreasing trend in the frequency of sleet and snow days. In summer, divergence between observations and reconstructions coincides with a reduction in network density prior to 1820, together with a period of uncertain data in key constituent series at Oxford and Kew. The trend to drier summers in observed *EWP*_*s*_ is not robust in our reconstructions with significance depending on start/end year and predictors used in model reconstructions.

There is much potential for further work. The summer findings warrant further research to better understand identified biases. Model reconstructions for spring and autumn are, perhaps surprisingly, relatively consistent with observations throughout the record. While there is some evidence for under‐catch of snow in the early autumn series, we find no such evidence for spring. We suggest that future research further interrogate the autumn and spring record.

Our findings challenge the widely reported trend to wetter winters and drier summers in England and Wales precipitation. Indeed, for winter the biases we identify in EWP are likely to have distorted many other long‐term European precipitation series (e.g., Murphy *et al.,* 2018) and dependent analyses (e.g., Wilby *et al.,* 2016), particularly through the under‐catch of snow. Our findings that pre‐1870 precipitation is likely too low in winter and that pre‐1820 precipitation is likely too high in summer raise issues about the application of early EWP data to historical drought analysis, climate model evaluation and for benchmarking other precipitation and proxy records for these periods. We recommend that such uses of observed EWP restrict application of the winter, autumn and annual series to post 1865 data, and summer to post 1820 data. For earlier periods, we advise use of our model reconstructions. Our work is a reminder of the importance of revisiting the quality of established long‐term records as new evidence and data sets become available.

## Supporting information


**Appendix S1.** Supporting Information.
