# Supplementary material for: Multi‐century trends to wetter winters and drier summers in the England and Wales precipitation series explained by observational and sampling bias in early records
Source: Int J Climatol. 2019 Jul 11;40(1):610–9. doi: 10.1002/joc.6208 (PMC6988466; doi:10.1002/joc.6208)
Supplement: Supplementary file 1 — Appendix S1. Supporting Information. [file JOC-40-610-s001.docx]

**Wetter winters and drier summers in England and Wales precipitation explained by observational and sampling bias in early records.**

Conor Murphy, Robert L. Wilby, Tom, K.R. Matthews, Peter Thorne, Ciaran Broderick, Rowan Fealy, Julia Hall, Shaun Harrigan, Phil Jones^,^ , Gerard McCarthy, Neil Macdonald, Simon Noone, Ciara Ryan

**Supporting Information**

**Table S1 List of stations that contribute to Manley’s London Sleet and Snow series, together with contributing dates, observer’s surname and altitude of the stations. The quality code applied to the data by Manley is as follows: A - data from first-class airfields and observatories; B – data from keen climatological observers; C – data from sites at which one daily visit was made to the instruments, and: D – data from stations overseen by observers who had other duties. In the Observer column the abbreviation Gents. Mag. refers to ‘The Gentleman’s Magazine’; MO is Meterological Office, and; RHS is Royal Horticultural Society.**

| **Station** | **Observer** | **Dates** | **Altitude (Feet)** | **Quality** |
| --- | --- | --- | --- | --- |
| Westminster | Gadbury | 1668-1700 | 30 | B |
| Lambeth | Ashmole | 1677-1685 | 30 | D |
| London | Downes | 1680-1694 | 50 | C |
| Ongar | Locke | 1692-1703 (Interrupted) | 250 | C |
| Upminster | Derham | 1697-1706 | 100 | C |
| W. Middlesex | Unknown | 1699-1717 | 100 | B |
| Richmond | Smith | 1713-1745 | 100 | D |
| London | Hauksbee | 1723-1728 | 50 | D- |
| London | Jurin | 1728-1750 | 50 | C |
| Tonbridge (Kent) | Hooker | 1728-1765 | 150 | C |
| London | Ayscough | 1755-1759 | 50 | C |
| London | Cuff | 1757-1761 | 50 | C |
| London area | Gents. Mag. | 1763-1781 | 50 | C- |
| Brentford | Hoy | 1771-1822 | 50 | C |
| London | Bent | 1785-1807 | 50 | B |
| Sunbury | Cowe | 1797-1839 | 50 | D- |
| London (Plaistow) | Howard | 1807-1830 | 100 | C |
| Stratford | Gibson | 1810-1827 | 100 | C |
| Greenwich | Belville | 1812-1856 | 100 | A- |
| Islington | Edwin | 1815-1866 | 100 | C to B+ |
| Epping | Squire | 1820-1856 | 300 | A- to B |
| London | Beaufort | 1821-1857 | 50 | C- |
| Chiswick | RHS | 1825-1869 | 50 | C- |
| Hackney | Underwood | 1832-1856 | 100 | C- |
| Greenwich | Royal Obs | 1841-1950 | 150 | A- to A |
| Clapham | Doxat | 1842-1879 | 100 | A- to B |
| Beckenham | Cator | 1857-1875 | 105 | B |
| Camden Sq | Symons | 1875-1899 | 110 | B+ |
| Kew Obs. | MO | 1876 (1902)-1960 | 18.5 | A- to A |

**Table S2 Predictors selected for each EWP_w_ model together with Pearson’s correlation of modelled (range from 1000 resamples and average from each model) Vs observed EWP_w_ for calibration (Cal), Evaluation (Eval) and reconstruction (Recon) periods. See Table 1 for predictor definitions.**

| **Model Number** | **Model**  **Structure** | **r Cal**  **(1900-2002)** | **r Eval**  **(1870-1899)** | **r Recon**  **(1767-1869)** |
| --- | --- | --- | --- | --- |
| 1 | LSLP+CET | 0.88 (0.80-0.93) | 0.90 (0.87-0.90) | 0.76 (0.69-0.78) |
| 2 | LSLP+PL | 0.90 (0.83-0.95) | 0.91 (0.88-0.91) | 0.78 (0.73-0.78) |
| 3 | LSLP+WI | 0.86 (0.75-0.93) | 0.77 (0.73-0.78) | 0.70 (0.59-0.73) |
| 4 | LSLP+KEOF | 0.87 (0.77-0.94) | 0.87 (0.82-0.87) | 0.72 (0.64-0.73) |
| 5 | LSLP+WI+CET | 0.89 (0.80-0.94) | 0.88 (0.81-0.90) | 0.78 (0.69-0.80) |
| 6 | KAVG | 0.86 (0.77-0.93) | 0.82 (0.82-0.82) | 0.55 (0.55-0.55) |
| 7 | KAVG+CET | 0.87 (0.74-0.93) | 0.85 (0.77-0.86) | 0.60 (0.48-0.66) |
| 8 | KAVG+PL | 0.87 (0.77-0.94) | 0.85 (0.80-0.86) | 0.62 (0.50-0.68) |
| 9 | G64+CET | 0.85 (0.74-0.91) | 0.86 (0.82-0.87) | 0.54 (0.44-0.60) |
| 10 | G64+PL | 0.86 (0.75-0.92) | 0.86 (0.82-0.86) | 0.59 (0.43-0.66) |
| 11 | G64+WI | 0.86 (0.75-0.93) | 0.83 (0.77-0.85) | 0.57 (0.48-0.63) |
| 12 | G65+CET | 0.88 (0.78-0.93) | 0.87 (0.81-0.87) | 0.59 (0.49-0.66) |
| 13 | G65+PL | 0.88 (0.81-0.92) | 0.86 (0.82-0.87) | 0.62 (0.49-0.68) |
| 14 | G65+WI | 0.87 (0.78-0.93) | 0.83 (0.78-0.84) | 0.58 (0.44-0.65) |
| 15 | G66+CET | 0.86 (0.77-0.92) | 0.84 (0.78-0.86) | 0.61 (0.52-0.67) |
| 16 | G66+PL | 0.87 (0.75-0.93) | 0.84 (0.78-0.86) | 0.63 (0.52-0.69) |
| 17 | G83+WI | 0.85 (0.74-0.90) | 0.74 (0.68-0.77) | 0.56 (0.47-0.61) |
| 18 | G83+WI+CET | 0.86 (0.78-0.91) | 0.80 (0.65-0.86) | 0.59 (0.47-0.65) |
| 19 | G84+CET | 0.86 (0.72-0.93) | 0.88 (0.85-0.89) | 0.57 (0.47-0.62) |
| 20 | G84+PL | 0.88 (0.79-0.93) | 0.89 (0.86-0.89) | 0.67 (0.57-0.70) |
| 21 | G84+KEOF | 0.85 (0.74-0.93) | 0.85 (0.81-0.85) | 0.56 (0.46-0.60) |
| 22 | G84+WI | 0.86 (0.75-0.93) | 0.74 (0.69-0.75) | 0.57 (0.45-0.62) |
| 23 | G84+WI+CET | 0.88 (0.82-0.93) | 0.84 (0.73-0.88) | 0.62 (0.52-0.68) |
| 24 | G84+KEOF+CET | 0.87 (0.77-0.93) | 0.89 (0.81-0.89) | 0.60 (0.45-0.65) |
| 25 | G85+CET | 0.87 (0.78-0.94) | 0.87 (0.84-0.87) | 0.58 (0.49-0.65) |
| 26 | G85+PL | 0.89 (0.80-0.93) | 0.89 (0.86-0.90) | 0.67 (0.59-0.71) |
| 27 | G85+WI | 0.86 (0.74-0.95) | 0.72 (0.69-0.73) | 0.55 (0.46-0.62) |
| 28 | G85+KEOF | 0.86 (0.75-0.93) | 0.83 (0.78-0.85) | 0.55 (0.43-0.60) |
| 29 | G85+WI+CET | 0.89 (0.80-0.95) | 0.84 (0.76-0.87) | 0.62 (0.47-0.69) |
| 30 | G86+CET | 0.85 (0.74-0.92) | 0.83 (0.76-0.84) | 0.58 (0.46-0.65) |
| 31 | G86+PL | 0.87 (0.79-0.94) | 0.86 (0.79-0.89) | 0.64 (0.53-0.69) |
| 32 | G87+PL | 0.84 (0.69-0.91) | 0.85 (0.77-0.87) | 0.64 (0.57-0.67) |
| 33 | G103+WI+CET | 0.85 (0.74-0.92) | 0.78 (0.68-0.85) | 0.57 (0.47-0.64) |
| 34 | G104+PL | 0.84 (0.71-0.91) | 0.90 (0.86-0.91) | 0.67 (0.58-0.70) |
| 35 | G105+PL | 0.84 (0.76-0.91) | 0.90 (0.84-0.91) | 0.65 (0.56-0.69) |

**Table S3 Predictors selected for each EWP_s_ model together with Pearson’s correlation of modelled (range from 1000 resamples and average from each model) Vs observed EWP_s_ for calibration (Cal), Evaluation (Eval) and reconstruction (Recon) periods. See Table 1 for predictor definitions.**

| **Model Number** | **Model**  **Structure** | **r Cal**  **(1900-2002)** | **r Eval**  **(1870-1899)** | **r Recon**  **(1767-1869)** |
| --- | --- | --- | --- | --- |
| 1 | LSLP | 0.87 (0.71-0.93) | 0.90 (0.90-0.90) | 0.77 (0.77-0.77) |
| 2 | LSLP+CET | 0.90 (0.81-0.94) | 0.90 (0.87-0.91) | 0.75 (0.68-0.77) |
| 3 | KAVG+CET | 0.83 (0.68-0.91) | 0.82 (0.80-0.82) | 0.60 (0.57-0.60) |
| 4 | G83+CET | 0.84 (0.69-0.92) | 0.90 (0.85-0.91) | 0.60 (0.57-0.60) |
| 5 | G83+CET+PL | 0.85 (0.73-0.91) | 0.90 (0.85-0.92) | 0.61 (0.57-0.61) |
| 6 | G84+CET | 0.86 (0.74-0.92) | 0.89 (0.85-0.90) | 0.62 (0.58-0.62) |
| 7 | G85+CET | 0.87 (0.75-0.94) | 0.87 (0.85-0.87) | 0.62 (0.59-0.62) |
| 8 | G86+CET | 0.86 (0.76-0.94) | 0.82 (0.77-0.83) | 0.60 (0.58-0.60) |

**Table S4 Predictors selected for modelling Kew [JJA], Oxford [JJA], precipitation together with Pearson’s correlation of modelled (median and range from 1000 resamples) Vs observed for calibration (Cal), Evaluation (Eval) and reconstruction (Recon) periods. See Table 1 for predictor definitions.**

| **Model** | **Model**  **Structure** | **r Cal**  **(1900-2002)** | **r Eval**  **(1870-1899)** | **r Recon**  **(1766-1869)** |
| --- | --- | --- | --- | --- |
| Kew JJA | LSLP+CET | 0.69 (0.48-0.82) | 0.73 (0.66-0.73) | 0.60 (0.48-0.61) |
| Oxford JJA | KAVG+CET | 0.66 (0.47-0.83) | 0.65 (0.47-0.71) | 0.51 (0.32-0.52) |

**Table S5 Predictors selected for modelling EWP Spring [MAM] together with Pearson’s correlation of modelled (median and range from 1000 resamples) Vs observed for calibration (Cal), Evaluation (Eval) and reconstruction (Recon) periods. See Table 1 for predictor definitions.**

| **Model Number** | **Model**  **Structure** | **r Cal**  **(1900-2002)** | **r Eval**  **(1870-1899)** | **r Recon**  **(1766-1869)** |
| --- | --- | --- | --- | --- |
| 1 | LSLP+PL | 0.81 (0.61-0.89) | 0.82 (0.80-0.82) | 0.61 (0.51-0.66) |
| 2 | KAVG+KEOF | 0.75 (0.54-0.86) | 0.70 (0.67-0.71) | 0.64 (0.58-0.64) |
| 3 | G65 | 0.78 (0.64-0.87) | 0.74 (0.74-0.74) | 0.64 (0.64-0.64) |
| 4 | G66 | 0.79 (0.67-0.91) | 0.72 (0.72-0.72) | 0.61 (0.61-0.61) |
| 5 | G65+PL | 0.79 (0.64-0.90) | 0.77 (0.73-0.78) | 0.62 (0.55-0.64) |
| 6 | G66+PL | 0.81 (0.64-0.90) | 0.74 (0.70-0.76) | 0.61 (0.56-0.61) |
| 7 | G85+PL | 0.80 (0.67-0.90) | 0.79 (0.76-0.79) | 0.57 (0.51-0.59) |

**Table S6 Predictors selected for modelling EWP Autumn [SON] together with Pearson’s correlation of modelled (median and range from 1000 resamples) Vs observed for calibration (Cal), Evaluation (Eval) and reconstruction (Recon) periods. See Table 1 for predictor definitions.**

| **Model Number** | **Model**  **Structure** | **r Cal**  **(1900-2002)** | **r Eval**  **(1870-1899)** | **r Recon**  **(1767-1869)** |
| --- | --- | --- | --- | --- |
| 1 | LSLP | 0.86 (0.70-0.93) | 0.81 (0.81-0.81) | 0.77 (0.77-0.77) |
| 2 | LSLP+KEOF | 0.87 (0.74-0.94) | 0.81 (0.76-0.81) | 0.79 (0.71-0.79) |
| 3 | LSLP+PL | 0.88 (0.73-0.95) | 0.80 (0.76-0.81) | 0.76 (0.69-0.78) |
| 4 | LSLP+CET+PL | 0.89 (0.76-0.95) | 0.80 (0.75-0.81) | 0.75 (0.68-0.79) |
| 5 | G65 | 0.82 (0.67-0.91) | 0.69 (0.69-0.69) | 0.65 (0.65-0.65) |
| 6 | G66 | 0.84 (0.70-0.91) | 0.69 (0.69-0.69) | 0.70 (0.70-0.70) |
| 7 | G67 | 0.80 (0.65-0.91) | 0.65 (0.65-0.65) | 0.70 (0.70-0.70) |
| 8 | G85 | 0.82 (0.66-0.82) | 0.76 (0.76-0.76) | 0.66 (0.66-0.66) |
| 9 | G84+PL | 0.85 (0.72-0.93) | 0.75 (0.67-0.77) | 0.63 (0.59-0.63) |
| 10 | G85+PL | 0.87 (0.71-0.93) | 0.74 (0.69-0.74) | 0.68 (0.64-0.69) |
| 11 | G86+PL | 0.87 (0.76-0.93) | 0.74 (0.67-0.76) | 0.68 (0.63-0.69) |
| 12 | G66+KEOF | 0.87 (0.71-0.94) | 0.75 (0.71-0.76) | 0.70 (0.65-0.70) |
| 13 | G67+KEOF | 0.85 (0.74-0.92) | 0.69 (0.66-0.70) | 0.66 (0.59-0.70) |
| 14 | G86+KEOF | 0.85 (0.70-0.93) | 0.70 (0.64-0.70) | 0.65 (0.56-0.70) |
| 15 | G84+CET+PL | 0.86 (0.74-0.92) | 0.75 (0.66-0.77) | 0.61 (0.57-0.63) |
| 16 | G87+CET+PL | 0.84 (0.73-0.92) | 0.70 (0.65-0.71) | 0.68 (0.61-0.70) |
| 17 | G87+CET+KEOF | 0.84 (0.69-0.91) | 0.77 (0.72-0.77) | 0.68 (0.58-0.71) |


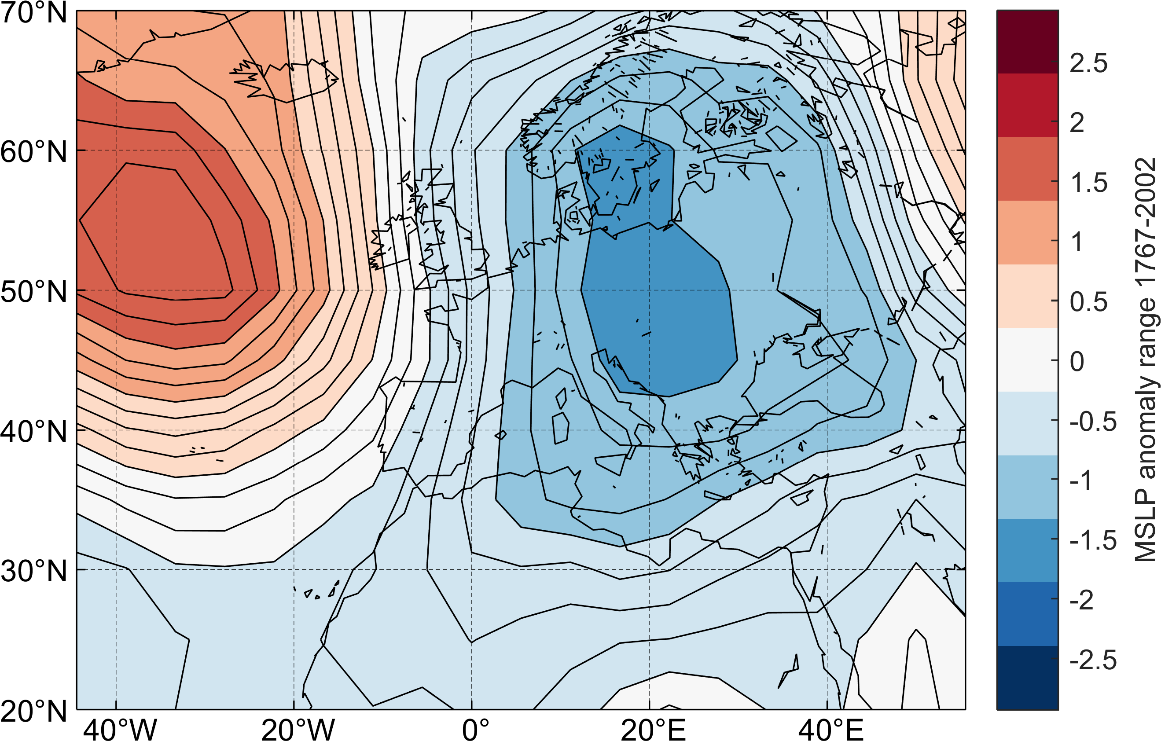


**Figure S1 Gridded winter [DJF] mean sea level pressure standardised anomalies for the thirty-year period 1791-1820. Anomalies are derived relative to mean SLP over the period 1767-2002.**


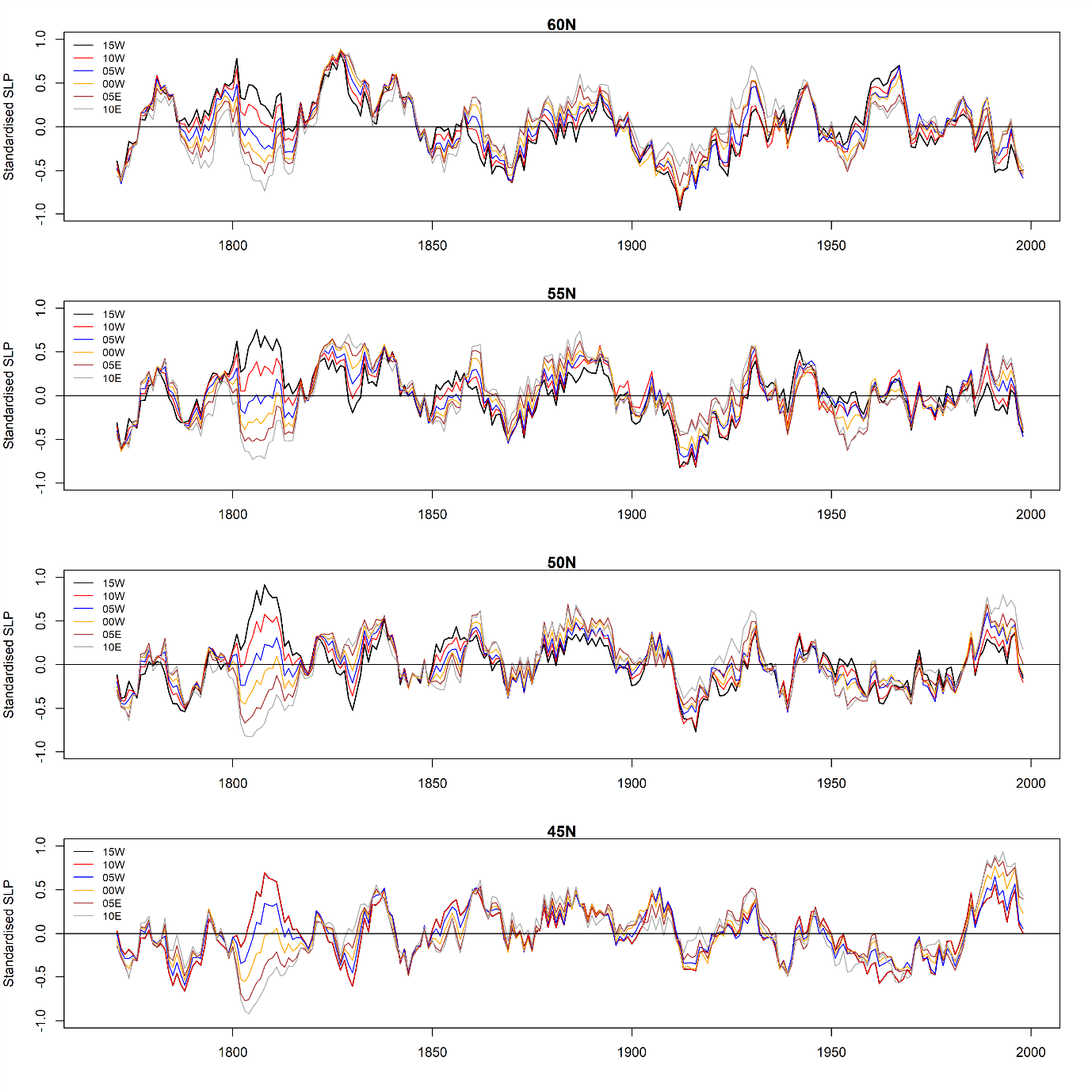


**Figure S2 Zonal transects (15^o^W-10^o^E) of standardised decadal rolling means of winter [DJF] Sea Level Pressure (SLP). Values for individual grids in the Küttel reconstructed dataset by latitude (45-60^o^N) for the period 1767-2002. Evident is the large zonal differences in winter SLP for the early 19^th^ Century, notable in the context of the entire record of reconstructions.**


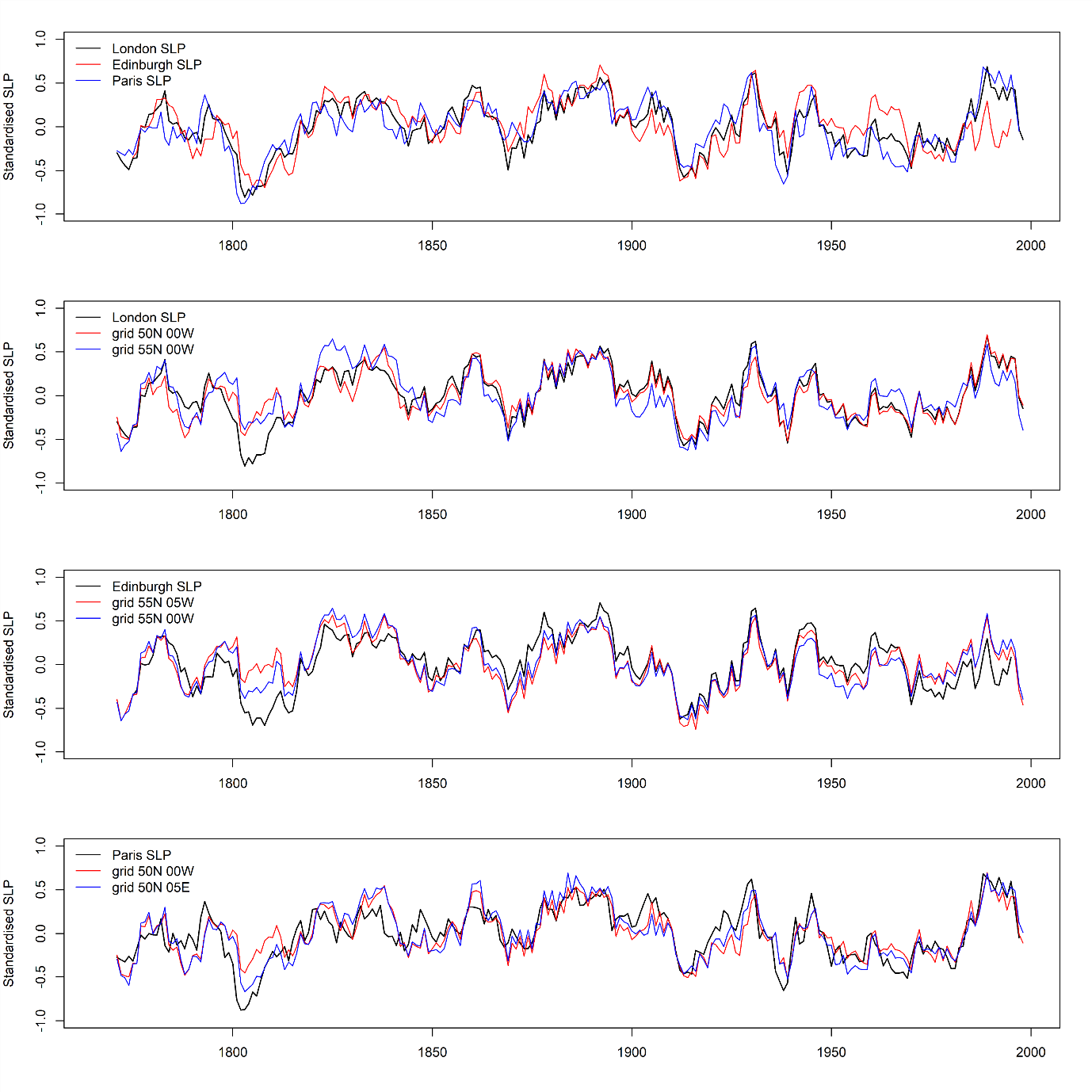


**Figure S3 Comparison of standardised decadal mean SLP for observed station based series at London, Edinburgh and Paris (top), together with comparisons of each station with the nearest 5^o^X5^o^ grid from the Küttel dataset. Evident is the difference between station-based SLP in the gridded reconstructions for the early part of the 19^th^ Century. SLP data for Paris and Edinburgh are used for comparison with gridded SLP reconstructions. Data for Paris have been quality assured (Cornes et al., 2012b) and were downloaded from the CRU website (**[**https://crudata.uea.ac.uk/cru/data/parislondon/**](https://crudata.uea.ac.uk/cru/data/parislondon/)**). Monthly SLP data for Edinburgh were retrieved from the ADVICE database (Jones et al., 1999) and downloaded from the KNMI Climate Explorer(**[**https://climexp.knmi.nl/geteuslp.cgi?id=someone@somewhere&WMO=6&STATION=EDINBURGH&extraargs**](https://climexp.knmi.nl/geteuslp.cgi?id=someone@somewhere&WMO=6&STATION=EDINBURGH&extraargs)**=).**

**
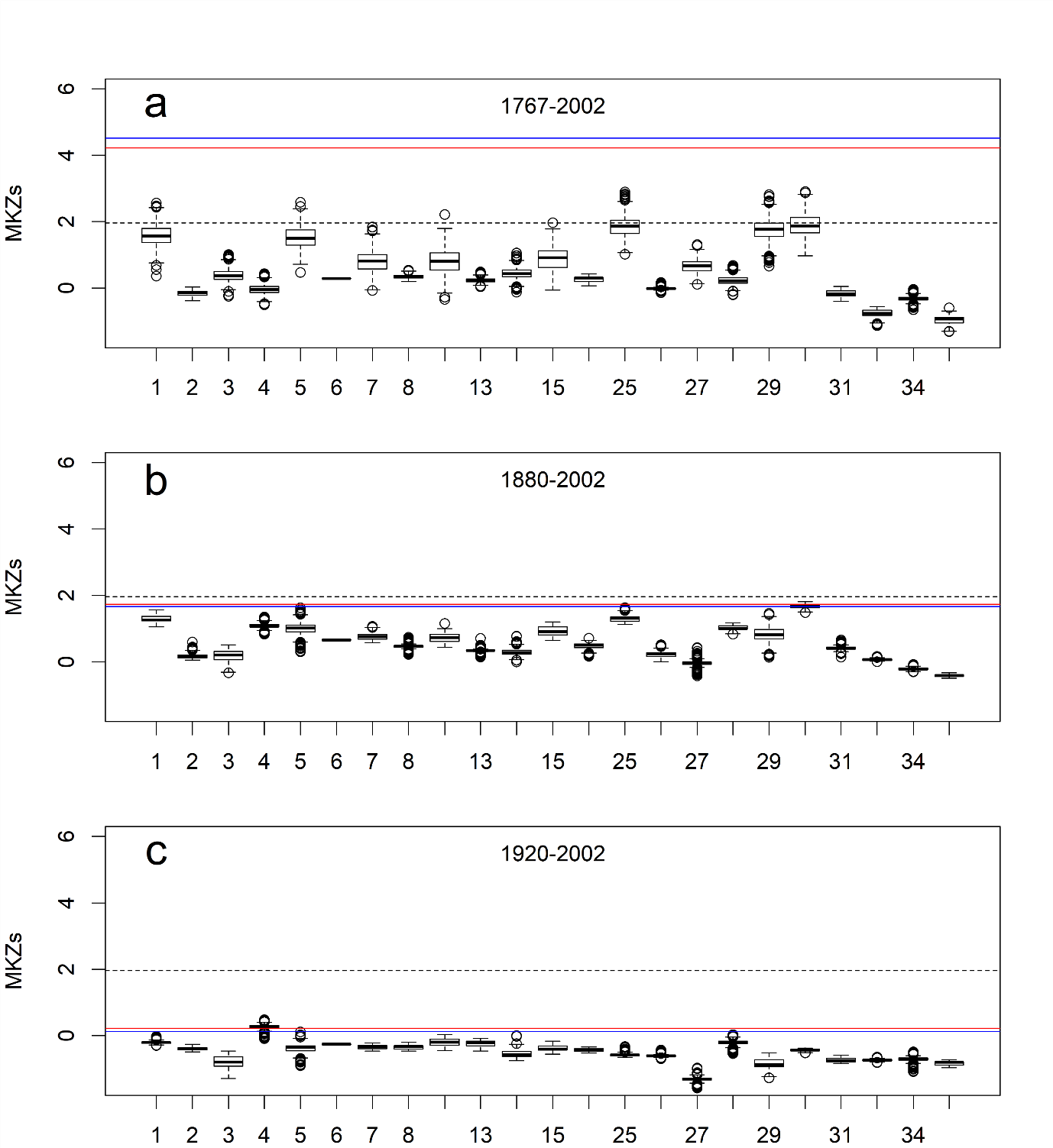
**

**Figure S4 Mann Kendall Zs values across 1000 re-samples of each regression model used to simulate EWP_w_. Trends are derived for three periods; a) 1767-2002; b) 1880-2002; and c) 1920-2002. Black dashed horizontal line represents the threshold for significant trend (0.05 level). The blue horizontal line represents the trend in observed EWP_w_ ending in 2002 and red horizontal line represents the trend in observed EWP_w_ ending in 2018.**


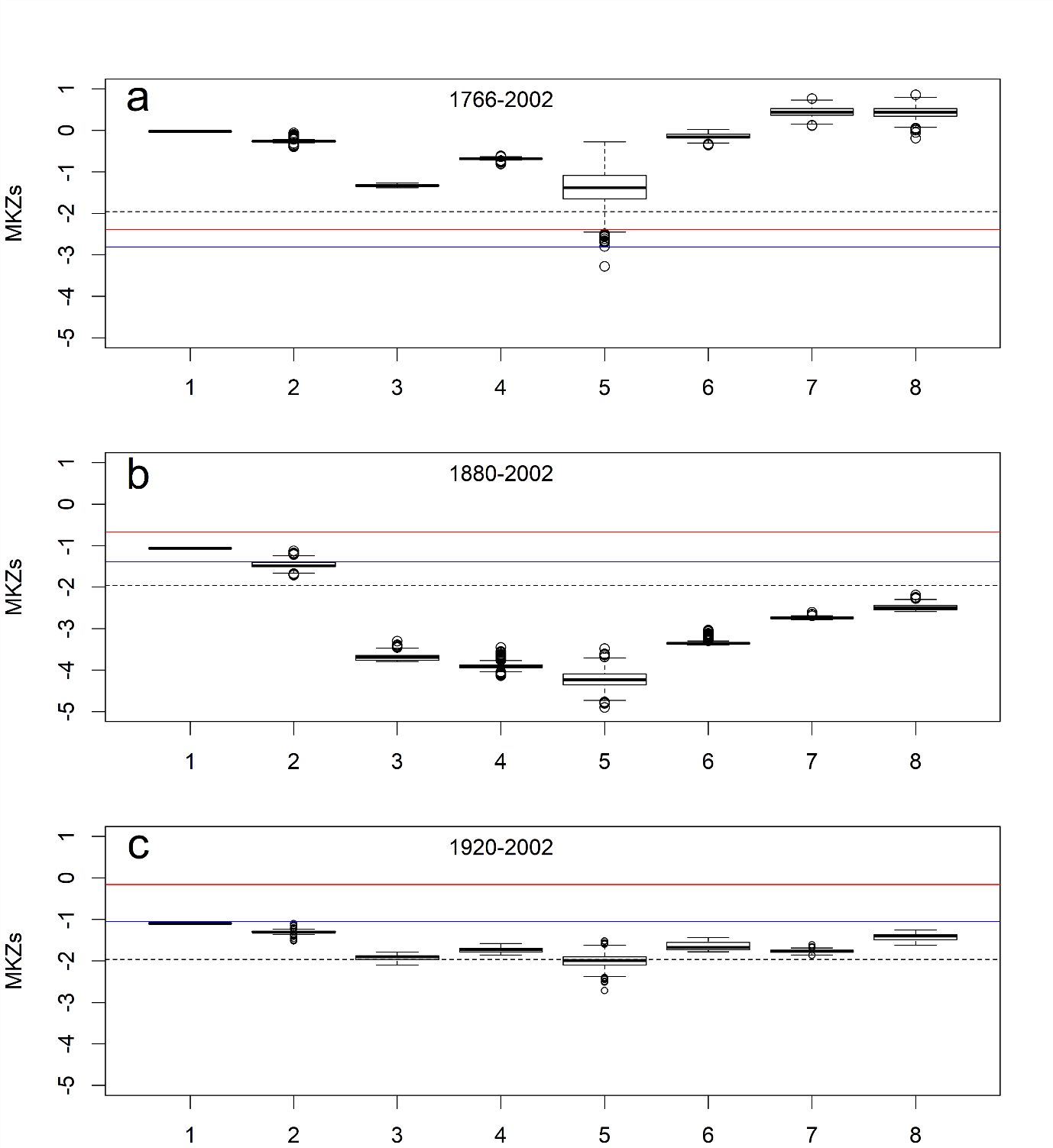


**Figure S5 Mann Kendall Zs values across 1000 re-samples of each regression model used to simulate EWP_s_. Trends are derived for three periods; a) 1767-2002; b) 1880-2002; and c) 1920-2002. Black dashed horizontal line represents the threshold for significant trend (0.05 level). The blue horizontal line represents the trend in observed EWP_w_ ending in 2002 and red horizontal line represents the trend in observed EWP_w_ ending in 2018.**
